# Proteomic Profile of Sperm in Infertile Males Reveals Changes in Metabolic Pathways

**DOI:** 10.1007/s10930-021-10013-w

**Published:** 2021-07-02

**Authors:** Jiaying Liang, Yichun Zheng, Weihong Zeng, Liuqing Chen, Shaofen Yang, Peng Du, Yujiang Wang, Xingsu Yu, Xiqian Zhang

**Affiliations:** 1 Reproductive Medical Center, Guangdong Women and Children Hospital, No. 521 Xingnan Road, Guangzhou, 511400 Guangdong China; 2 Children Inherit Metabolism and Endocrine Department, Guangdong Women and Children Hospital, Guangzhou, 511400 China

**Keywords:** Infertility, iTRAQ, Mass spectrometry, Sperm

## Abstract

The objective of the present study was to investigate the differences in the proteomic profiles of sperm from infertile males with severe oligoasthenoteratozoospermia requiring intracytoplasmic sperm injection (ICSI) and normal control sperm from fertile males. Isobaric tag for relative and absolute quantitation labeling and liquid chromatography–tandem mass spectrometry was performed for identifying proteins in the sperm of infertile and fertile males. Differentially expressed proteins were analyzed via the Gene Ontology and Kyoto Encyclopedia of Genes and Genomes (KEGG) databases through the Database for Annotation, Visualization, and Integrated Discovery, and protein–protein networks were produced using the Search Tool for Retrieval of Interacting Genes. Immunofluorescence and western blotting verified the differential expression of Y-box-binding protein 1(YBX1), adenylate kinase 1 (AK1), and aconitase 2, mitochondrial (ACO2) proteins. Altogether, 3444 proteins were identified in the sperm of infertile and fertile males, and 938 were differentially expressed between the two groups. Pairwise comparisons revealed that 226 and 712 proteins were significantly upregulated and downregulated in infertile males, respectively. These proteins were significantly enriched in metabolic pathways as per KEGG enrichment analysis. YBX1 expression was upregulated in the sperm heads of patients requiring ICSI treatment, whereas AK1 and ACO2, which are critical enzymes involved in energy metabolism, were downregulated in the sperm tails of the same patients. This result indicates that metabolism may have a crucial role in maintaining normal sperm function. Overall, our results provide insights that will further help in investigating the pathogenic mechanisms of infertility and possible therapeutic strategies.

**Supplementary Information:**

The online version contains supplementary material available at 10.1007/s10930-021-10013-w.

## Introduction

Approximately 15% of couples of childbearing age experience infertility, 50% of which is due to factors in males [1]. Although the cause of male infertility is complex, genetic factors, smoking, varicocele, obesity, and spinal cord injury are among the most common contributing factors [2]. Clinically, male infertility assessment is based on semen analysis of sperm motility and morphology. Sperm motility is the most critical factor for normal fertilization because impaired sperm account for over 80% cases of male infertility. Sperm morphology, which affects sperm motility, is the second most crucial contributing factor for male fertility. Infertile males often present with abnormal morphology of the sperm head, neck, body, or tail [3].

Analysis based on sperm motility and morphology is clinically important for assessing male fertility; however, proteomic analysis of sperm from infertile men can reveal more information. For instance, the sperm of infertile men with asthenozoospermia and oligoasthenozoospermia exhibit downregulated expression of seminogelin II precursor and clusterin isoform 1 [4, 5]. Using high-throughput isobaric tag for relative and absolute quantification (iTRAQ) proteomic technology, aberrant expression of proteins involved in metabolism, transport, antioxidation, and immune response has been identified in oligoasthenozoospermic seminal plasma [6]. In addition, iTRAQ-based analysis of sperm proteomics has revealed aberrant expression of sperm proteins in normozoospermic men who required rescue-intracytoplasmic sperm injection (ICSI, which is a routine assisted reproductive technology for infertile men) to achieve pregnancy after the failure of conventional in vitro fertilization [7, 8]. These findings reveal the importance of proteomic analysis in characterizing the sperm quality of infertile men. These studies provide data that can facilitate further investigation into the pathogenic mechanisms underlying male infertility. However, although changes in some proteins involved in sperm motility and morphology have been reported, a comprehensive profile is still lacking, particularly in males with oligoasthenoteratozoospermia requiring ICSI. Furthermore, in infertile men who require ICSI, a suitable biomarker indicative of the benefits of the treatment remains unavailable. Therefore, further studies on the expression levels and functions of proteins in the sperm of infertile males should be conducted.

iTRAQ is a well-established high-throughput technology used for global proteomic profiling [9]. In the present study, we selected infertile men diagnosed with severe oligoasthenoteratozoospermia in China and compared their sperm protein profiles with those of fertile men from the same population. Our research aims were to elucidate the proteomic profile of sperm from infertile men, provide novel insights into potential biomarkers, and provide a theoretical basis upon which the diagnosis and treatment of male infertility can be based.

## Materials and Methods

### Study Subjects

12 infertile male patients with severe oligozoospermia, asthenozoospermia, oligoasthenozoospermia, teratozoospermia, oligoasthenoteratozoospermia, or acrosome dysfunction who required ICSI treatment at Guangdong Maternal and Child Health Hospital and 12 healthy male volunteers (husbands of normal pregnant women) with normozoospermia [i.e., normal control (NC)] were recruited. According to the “WHO laboratory manual for the examination and processing of human semen” (WHO 5th Edition) [10], normozoospermia is categorized as follows: concentration ≥ 15 × 10^6^/mL, progressive motility (PR) sperm percentage ≥ 32%, and normal morphological sperm percentage ≥ 4%. According to the clinical operating procedures of the study center, the inclusion criteria of the ICSI group were as follows: total PR sperm ≤ 5 × 10^6^/mL, abnormal acrosome function (acrosome reaction following ionophore challenge ≤ 3%), or abnormal sperm morphology (percentage of sperm with normal morphology ≤ 1%). Exclusion criteria included the following: (1) genetic diseases such as abnormal karyotypes and Y chromosome microdeletion; (2) systemic diseases and congenital diseases; (3) infectious diseases and reproductive tract infections; (4) testicular trauma, cryptorchidism, and other surgical history; and (5) long-term exposure to drugs or toxic substances. The ethics committee of Guangdong Women and Children Hospital approved this study (approval number: 202001159), and all participants provided written informed consent.

### Sperm Collection

Fresh sperm were collected from the study subjects by masturbation after a minimum of 3 days of abstinence. The semen was liquefied for 30 min before being isolated using SpermGrad™ (Vitrolife, CO, USA). Purified sperm were washed three times with a sperm washing solution (SpermRinse™; Vitrolife) and then evaluated for sperm concentration, motility, viability, and morphology according to the WHO laboratory manual for the examination and processing of human semen (WHO 2010 guidelines) [10]. Subsequently, the sperm were subjected to proteomic analysis.

### Sample Preparation and iTRAQ Labeling

We prepared the samples according to previously reported methods [11]. We randomly selected two sperm samples from the oligoasthenoteratozoospermic men and two control samples from the healthy subjects. Sperm were dissolved in a 7-M urea, 2-M thiourea, 65-mM DTT, and 1% (v/v) protease inhibitor mixture. Total protein (300 µg) from each sample was processed by reduction, alkylation, and trypsinization (Promega, Madison, WI). The digested samples were reconstituted in 50 µL of 0.5-M triethylammonium bicarbonate. Labeling was performed using an iTRAQ kit per the manufacturer’s protocol (AB Sciex, Foster City, California, USA) [12]. The sperm samples of NCs were labeled with iTRAQ tags 114 and 115, whereas those of patients with ICSI were labeled with tags 117 and 119. The labeled samples were dried in a rotary vacuum concentrator before further analysis.

### Two-Dimensional Liquid Chromatography–Tandem Mass Spectrometry (LC–MS/MS)

The iTRAQ-labeled and mixed peptides were fractionated using a high-performance liquid chromatography system with a strong cation exchange column (Dionex, Sunnyvale, CA). The peptides were separated using a linear gradient composed of mobile phase A and mobile phase B. Buffer A included H_2_O:acetonitrile (ACN) (95:5) with 5-mM ammonium formate (pH 2.7); a flow rate of 200 µL/min was used for peptide separation in buffer A. Buffer B was composed of buffer A with 800-mM ammonium formate (pH 2.7). The separation gradient was as follows: 0–5 min, 5% B; 5–10 min, 5–10% B; 10–60 min, 10–40% B; 60–65 min, 40–95% B; 65–75 min, held at 95% B; and 75–85 min, 95–5% B until the next run. A fraction of 100 µL was collected every minute, and 20 fractions were collected in total. Ultraviolet detection was set at 214/280 nm. The peptides were vacuum-dried and resuspended in 50 µL of dissolving solution (5% ACN and 0.1% formic acid). They were then eluted in a gradient of buffer A (0.1% formic acid) to buffer B (80% ACN containing 0.1% formic acid) at a rate of 300 nL/min for 65 min. Tandem mass spectrometry (MS/MS) analysis was performed on a Q Exactive system (Thermo Fisher Scientific, Waltham, MA, USA) using the information-dependent acquisition mode. The MS parameters were as follows: scan range = 350–1800 m/z; resolution = 70,000; maximum injection time per mass spectrum = 100 ms. Tandem mass spectra were recorded at a resolution of 17,500. The 20 strongest precursors were selected for fragment separation per MS cycle. The maximum injection time was 60 ms.

### LC–MS/MS Database Search

The output of raw files from LC–MS/MS analysis was converted to MGF format using Proteome Discoverer 1.4 (Version 1.4.0.288, Thermo Fisher). The mass spectra files in MGF format and protein search libraries were then imported into ProteinPilot™ Software 4.5 (version 1656, AB Sciex) for database searching. The relative expression of the protein was calculated based on the ratio between two reporter ions of the same peptides (117:114 or 119:114; 117:115 or 119:115). A protein was considered differentially expressed if it met the following criteria: unused ProtScore > 1.30 (> 95% confidence), coefficient of variation ≤ 0.5, average (AVG) ≥ 1.5 or AVG ≤ 0.67, P-value using *t* test < 0.05, peptides ≥ 2 (> 95% confidence), and false discovery rate (FDR) < 0.01.

The Gene Cluster 3.0/TreeView software was used to visualize the differential expression and relationships among protein groups based on the mean relative volume of each protein spot to analyze the differentially expressed proteins. These differentially expressed proteins were subjected to Gene Ontology (GO) analysis to assess for related cell components, molecular function, and biological process terms. Kyoto Encyclopedia of Genes and Genomes (KEGG) analysis was also performed based on the differential expression of proteins. GO and KEGG analyses were conducted through the Database for Annotation, Visualization, and Integrated Discovery (https://david.ncifcrf.gov/). FDR ≤ 0.001 was set as the threshold for significant enrichment. In addition, protein–protein interaction (PPI) networks were produced using the Search Tool for Retrieval of Interacting Genes (https://string-db.org) and visualized using Cytoscape.

### Immunofluorescence Assay

Sperm cells were centrifuged, resuspended in phosphate-buffered saline, and added to polylysine-coated coverslips. After air-drying, the coverslips were fixed in 4% formaldehyde for 20 min and then blocked using fetal bovine serum for 1 h at room temperature. Either ACO2 antibody (Proteintech, IL, USA), YBX1 antibody (Proteintech) or AK1 antibody (Santa Cruz, CA, USA) was added to the coverslips and incubated at 4 °C overnight. A secondary antibody labeled with fluorescein isothiocyanate was added and incubated for 1 h at room temperature. Images were obtained using an inverted fluorescence microscope (Olympus, Tokyo, Japan).

### Western Blot

A BCA Protein Assay Kit (Pierce, MA, USA) was used to quantify the total protein concentration extracted from sperm. From each sample, 30 µg of total protein was separated by 10% sodium dodecyl sulfate polyacrylamide gels and transferred to polyvinylidene fluoride membranes (Millipore, MA, USA). The membranes were blocked with 5% non-fat milk for 1 h followed by incubation in ACO2 antibody (Proteintech), YBX1 antibody (Proteintech), AK1 antibody (Santa Cruz), or GAPDH antibody (Proteintech) overnight at 4 °C. The following day, a horseradish peroxidase-conjugated secondary antibody (Jackson ImmunoResearch, West Grove, PA, USA) was added to the membranes. The expression of proteins on each membrane was determined using an ECL western blotting Kit (Amercontrol Biosciences, London, UK).

## Results

### Overview of the Protein Expression Profile in Sperm

**Fig. 1:**
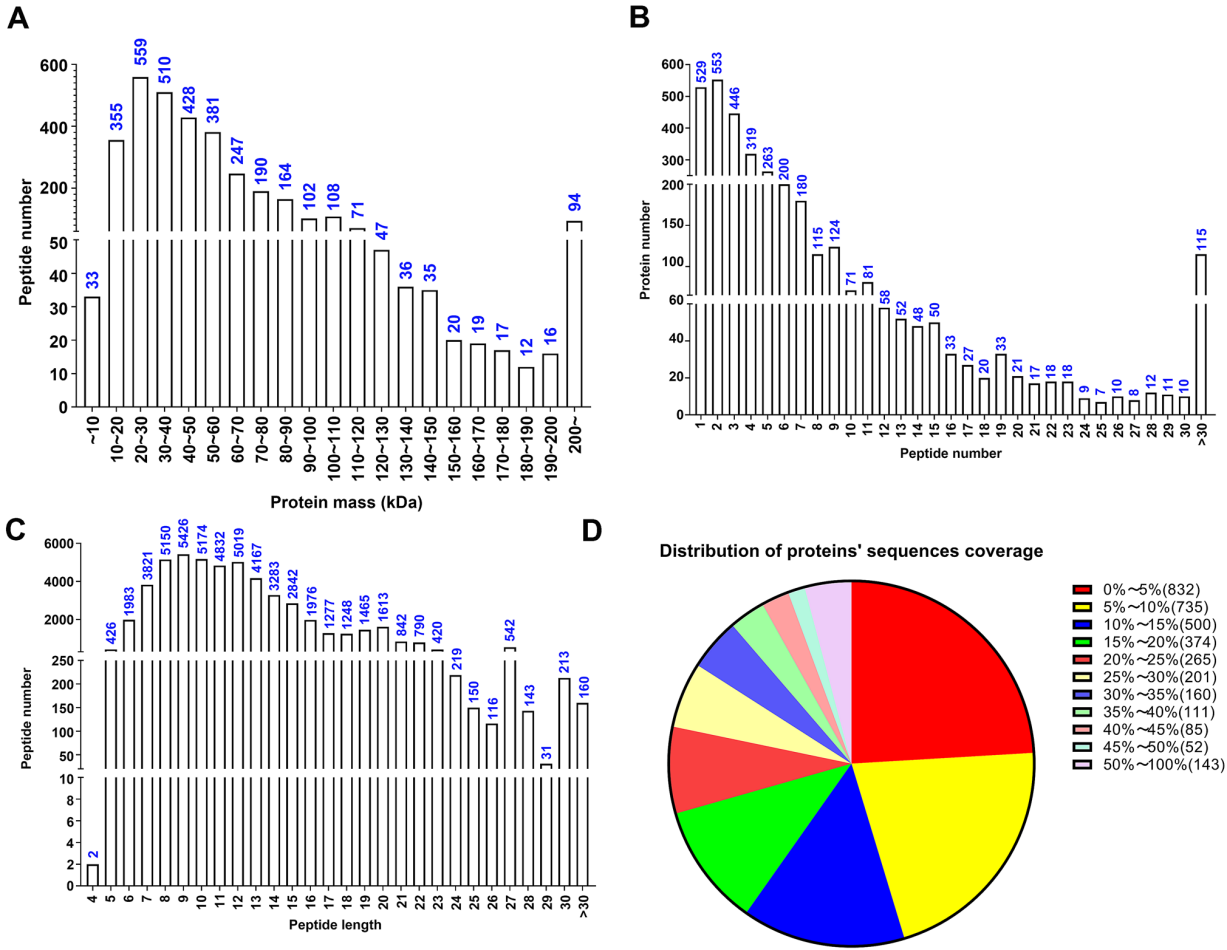
Proteomic analysis of sperm samples. Overview of the protein mass (**A**), number of peptides (**B**), peptide length (**C**), and protein sequence coverage (**D**) identified by the iTRAQ and LC–MS/MS analysis. iTRAQ: isobaric tag for relative and absolute quantification; *LC*–*MS*/*MS* liquid chromatography and MS/MS analysis

First, we explored the overall protein expression profiles in the fertile (12 samples from NC) and infertile (12 samples from males requiring ICSI) sperm groups (Table 1). Specifically, two NC samples (114 and 115) and two ICSI samples (117 and 119) were selected for proteomic analysis by MS. Altogether, 3444 proteins were detected in the sperm samples of both groups. The molecular weight of the proteins was 20–90 kD (Fig. 1A). The largest proportion of proteins were identified by 2 peptides per protein followed by 1, 3, 4, 5, and 6 peptides per protein, which corresponded to 553, 529, 446, 319, 263, and 200 proteins in each category (Fig. 1B). The length of the peptides ranged from 7 to 15 amino acids (Fig. 1C). The sequence coverage of most proteins was 0–15%, whereas the sequence coverage of 143 proteins reached 50–100% (Fig. 1D).

**Table 1:** Parameters of sperm in infertile ICSI patients and normal fertile males

	Sperm parameters							Female parameters				
Sample	Age (year)	Concentration (10^6^/ml)	Morphology* (%)	PR%	PR (*10^6^)	DFI (%)	AR (%)	Age (year)	Infertility (Year)	Fertilization (2PN/OOC)	High quality embryos	Pregnancy# (Y/N)
P1 (teratozoospermia and asthenozoospermia)	26	20.2	0.49	11.9	8.40	25.2	6	24	2	9/22	3	Y
P2 (oligoasthenozoospermia and teratozoospermia)	37	3.2	2.30	36.8	2.76	NA	NA	NA	NA	NA	NA	NA
P3 (teratozoospermia and oligoasthenozoospermia)	28	12.8	0.96	16.5	7.14	25.0	NA	NA	NA	NA	NA	NA
P4 (oligoasthenoteratozoospermia)	31	3.5	0.97	25.0	2.25	NA	NA	NA	NA	NA	NA	NA
P5 (oligoasthenoteratozoospermia and acrosome dysfunction)	33	1.7	0.97	60.0	2.24	23.2	3	25	4	20/23	15	Y
P6 (oligoasthenoteratozoospermia)	28	0.8	0.49	20.0	0.66	30.0	4	29	2	13/16	8	Y
P7 (oligoasthenozoospermia, acrosome dysfunction and teratozoospermia)	32	2.1	2.36	25.6	1.60	7.8	2	31	10	5/6	2	Y
P8 (oligoasthenozoospermia, acrosome dysfunction and teratozoospermia)	33	33.2	3.23	2.1	0.84	30.6	3	NA	NA	NA	NA	NA
P9 (oligoasthenoteratozoospermia and acrosome dysfunction)	37	0.6	0.00	18.5	0.10	24.8	2	34	5	10/16	7	N
P10 (teratozoospermia and asthenozoospermia)	38	18.0	0.95	26.4	11.75	26.6	NA	38	13	6/10	5	Y
P11 (teratozoospermia and oligozoospermia)	32	11.8	1.18	50.0	8.85	22.6	6	30	1	4/6	2	N
P12 (teratozoospermia and oligoasthenozoospermia)	33	13	0.98	24.0	9.98	20.8	4	33	3	7/12	4	Y
N1	28	52.4	2.00	73.4	88.55	16.2	5					
N2	37	96.2	4.85	70.8	170.50	24.0	8					
N3	43	49.9	0.96	32.3	37.03	27.8	8					
N4	43	130.1	1.32	68.1	230.62	NA	7					
N5	33	60.0	9.80	48.0	28.80	NA	NA					
N6	31	60.0	9.80	48.0	57.60	NA	NA					
N7	32	60.0	9.80	48.0	28.80	NA	NA					
N8	38	90.0	5.00	38.0	34.20	NA	NA					
N9	34	68.0	9.80	42.0	28.56	NA	NA					
N10	38	72.0	5.00	44.0	31.68	NA	NA					
N11	36	70.0	10.60	44.0	30.80	NA	NA					
N12	35	75.0	11.40	42.0	31.50	NA	NA					

*P* infertile male, *N* normal fertile male

*Percentage of normal sperm; # Outcome of ICSI; *PR* progressive motility, *DFI* sperm DNA fragmentation index, *AR* acrosome reaction after ionophore challenge; *PN* pronuclei, *Ooc* oocytes, *NA* not applicable

### Differentially Expressed Proteins Identified in the Sperm of Infertile Males who Received ICSI

**Fig. 2:**
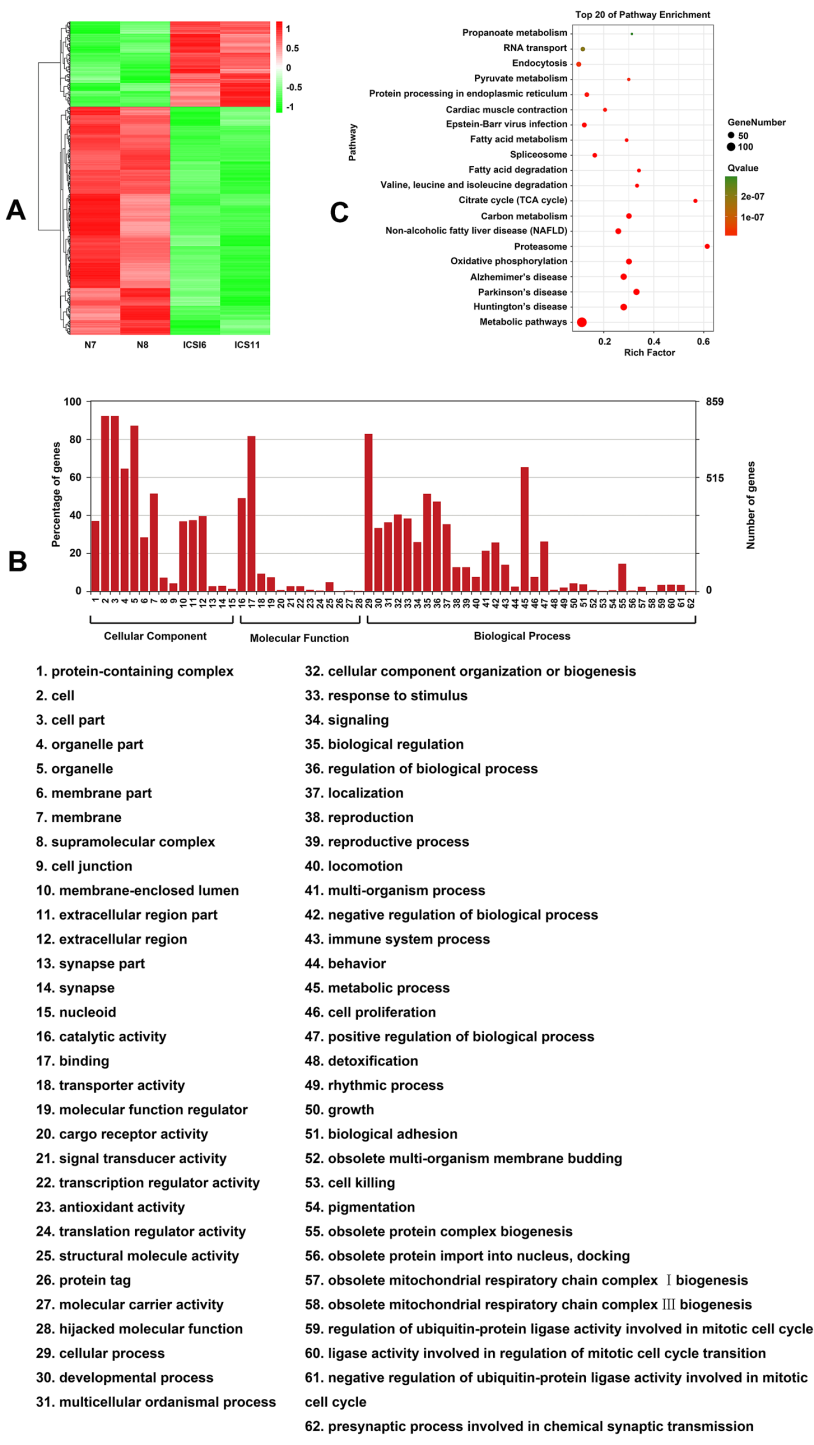
Differentially expressed proteins in the infertile males who received ICSI, identified by the iTRAQ and LC–MS/MS. **A** Heatmap showing the differentially expressed proteins in infertile males relative to their expression in normal fertile males. Samples and proteins were clustered based on the similarity of the expression profiles of proteins. The dendrogram indicates the similarity between the samples. GO (**B**) and KEGG (**C**) analyses show that the differentially expressed proteins are associated with multiple terms and pathways. *NC* normal control, *ICSI* intracytoplasmic sperm injection, *iTRAQ* isobaric tag for relative and absolute quantification, *LC*–*MS*/*MS* liquid chromatography and MS/MS analysis, *GO *Gene Ontology, *KEGG* Kyoto Encyclopedia of Genes and Genomes

We further investigated the proteins that were differentially expressed between the ICSI and NC groups. The protein expression profile of sperm from the ICSI group differed from that of the NC group (Supplementary Table 1). A heatmap revealed 938 differentially expressed proteins, from which 712 and 226 were significantly downregulated and upregulated, respectively, in the ICSI group relative to the NC group (Fig. 2A). GO enrichment analysis showed that these differentially expressed proteins were significantly enriched in the cell, cell parts, membrane parts, binding, cellular process, and metabolic process GO terms (Fig. 2B). Top 20 KEGG pathway enrichment analyses also showed that these differentially expressed proteins were significantly enriched in signaling pathways such as metabolic pathways and oxidative phosphorylation (Fig. 2C).

**Fig. 3:**
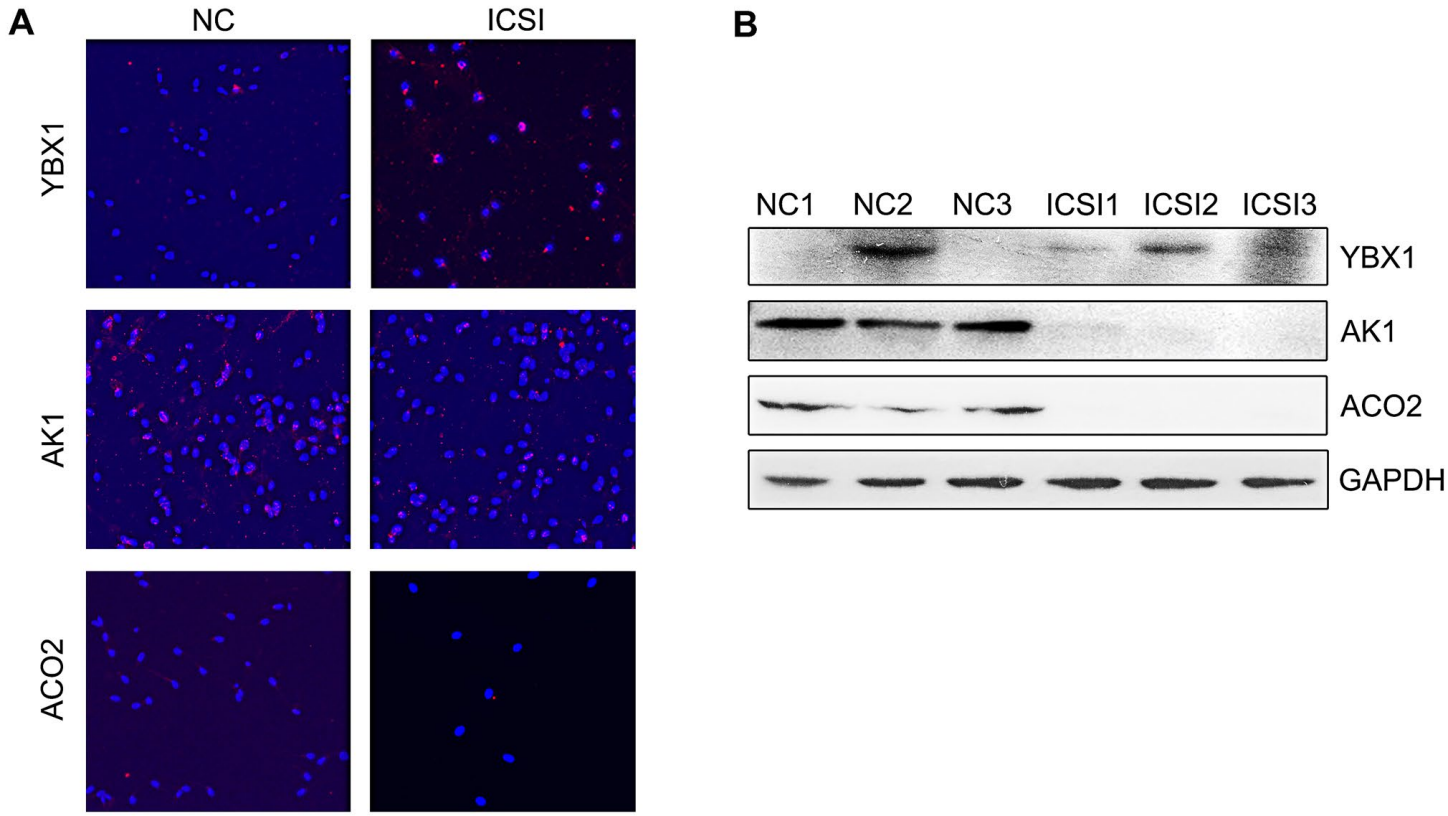
Upregulation of YBX1 and downregulation of AK1 and ACO2 were experimentally validated by immunofluorescence (**A**) and western blotting (**B**). *NC* normal control, *ICSI* intracytoplasmic sperm injection, *ACO2* aconitase 2, mitochondrial, *AK1* adenylate kinase 1, *YBX1* nuclease-sensitive element-binding protein 1

**Fig. 4:**
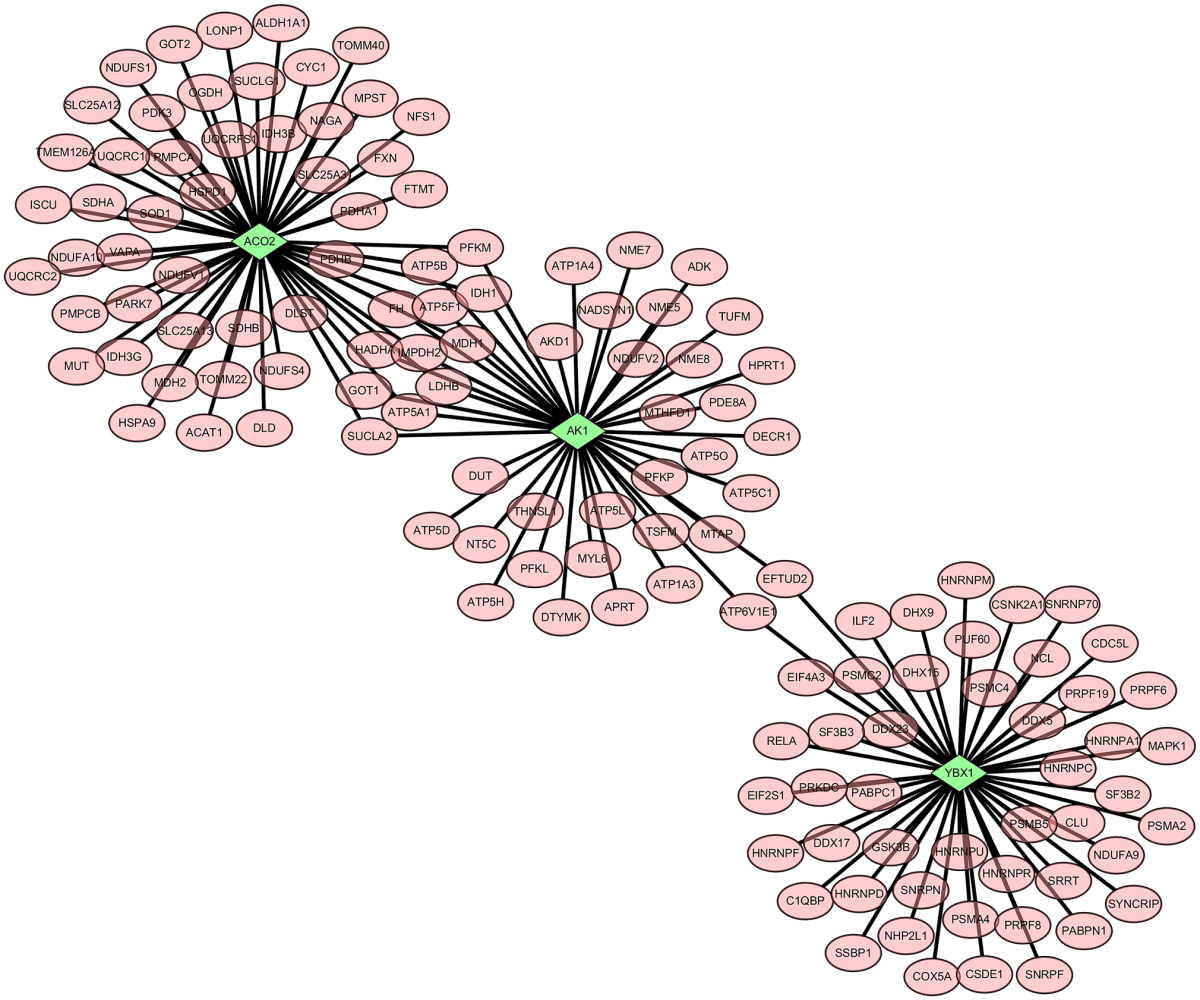
Protein–protein interaction network of YBX1, AK1, and ACO2. *ACO2* aconitase 2, mitochondrial, *AK1* adenylate kinase 1, *YBX1* nuclease-sensitive element-binding protein 1

Based on the abovementioned results and previous studies [13–16], we selected three significantly differentially expressed proteins, including ACO2, AK1, and YBX1, for further validation. These proteins are essential for sperm motility and spermatogenesis. YBX1, which is also known as Y-box transcription factor, is involved in spermatogenesis [15, 16]. Mitochondrial ACO2 and AK1 are required for sperm motility [13, 17]. Consistent with the proteomic analysis results (Table 2; Supplementary Table 1), the upregulation of YBX1 and downregulation of ACO2 and AK1 in sperm from the ICSI group were experimentally validated (Fig. 3A and B). ACO2 and AK1 are metabolic enzymes that are essential for energy metabolism, whereas YBX1 functions both as a DNA- and RNA-binding protein and has been implicated in numerous cellular processes, including regulation of transcription and translation, pre-mRNA splicing, DNA repair, and mRNA packaging [18, 19]. The PPI networks of ACO2, AK1, and YBX1 were also constructed (Fig. 4); these networks were relatively independent but connected to ATP and metabolism-associated proteins such as solute carrier family 25 member 3 (SLC25A3), fumarate hydratase (FH), and mitogen-activated protein kinase 1 (MAPK1).

**Table 2:** Information on the candidate differentially expressed proteins

Protein	117:114*	119:114*	117:115*	119:115*	T.TEST	Change	Pathway
ACO2	0.14	0.18	0.22	0.27	8.75E-05	DOWN	Catalyzes the interconversion of citrate to isocitrate via cis-aconitate in the second step of the citrate cycle (TCA cycle); energy metabolism.
AK1	0.3	0.18	0.24	0.14	1.92E-04	DOWN	Energy metabolism and homeostasis of cellular adenine nucleotide ratios
YBX1	4.09	2.88	2.65	1.87	0.026	UP	Regulation of transcription and translation, pre-mRNA splicing, DNA reparation and mRNA packaging

*Indicates intracytoplasmic sperm injection compared with normal control; *ACO2* aconitase 2, mitochondrial, *AK1* adenylate kinase 1, *YBX1* nuclease-sensitive element-binding protein 1

## Discussion

In the present study, a proteomic analysis of sperm from male infertile patients with severe oligozoospermia, asthenozoospermia, or teratozoospermia who required ICSI treatment was conducted. Compared with normal fertile men, 938 proteins were differentially expressed in the infertile patients. GO and KEGG analyses revealed that these differentially expressed proteins were associated with metabolic pathways. Moreover, we experimentally validated the downregulation of ACO2 and AK1 and the upregulation of YBX1 in sperm from infertile men.

Previous studies on patients with infertility have shown differential expression of proteins involved in posttranslational modifications associated with non-sperm morphology, vitality, and sperm volume [2]. Proteomic studies on patients with lower sperm volume have also been reported [5]. In contrast to the subjects used in previous research, the subjects in this study were patients with infertility having low sperm volume, low viability, and abnormal morphology who required ICSI. In addition, the iTRAQ-based proteomic technology that offers improved accuracy in terms of protein quantification was used. The methods used to screen differentially expressed proteins in the two previous studies included LC–MS/MS, two-dimensional gel electrophoresis, liquid chromatography–mass spectrometry, and matrix-assisted laser desorption ionization–time-of-flight-mass spectrometry analysis; however, the iTRAQ-based technology used here is a high-throughput method used extensively for quantitative proteomic analysis of sperm [7, 20, 21]. Therefore, as expected, additional differentially expressed proteins that are potentially associated with infertility were discovered.

Previous studies have identified the functions of some differentially expressed proteins in sperm samples from infertile men. For instance, HSPA2 contributes to sperm and egg recognition because it binds to the oocyte zona pellucida; thus, low expression of HSPA2 indicates defective sperm recognition [22]. BAG6 maintains HSPA2 stability; the loss of HSPA2 and BAG6 occurs in sperm that fail to bind to the oocyte zona pellucida [23]. BAG6 is a protein that plays an important role in fertility. It is often underexpressed in patients with infertility, including those with oligospermia [22, 24, 25]. The protein SPA17 may also regulate the recognition and binding to the oocyte zona pellucida [26, 27].

Interestingly, in previous studies, downregulation of BAG6 was reported in patients with infertility, but no differences in HSPA2 and SPA17 expression were observed between patients with infertility and subjects with normal fertility [2]. In our protein profile, HSPA2, BAG6, and SPA17 were all shown to be downregulated. This may be attributable to the patients in this study having less sperm count, lower motility, and abnormal sperm morphology. Therefore, the downregulation of HSPA2, BAG6, and SPA17 in the present study indicates that sperm and oocyte recognition may be associated with the abnormal sperm linked with infertility. However, this speculation requires further investigation.

Compared with other proteomics studies of patients with oligospermia and weak sperm, more differentially expressed proteins were found in the resent study. However, SGII and clusterin isoform 1, which were previously reported in patients with oligospermia, were not found to be downregulated [5]. Although results of this study did not show downregulation of ODF2 compared with patients with asthenozoospermia, ODF3 expression was noted to be downregulated, which belongs to the same family as ODF2. Because ODF is a component of flagella and helps retain the elastic stiffness of sperm flagella, the low expression of ODF3 may indicate a decrease in the sperm motility of patients [28]. Consistent with findings from patients with asthenospermia, differential expression of metabolic pathway-related proteins was also observed in our study. For instance, COX6B, which is involved in ATP synthesis [29], was also downregulated in patients with asthenospermia. In addition, the upregulation of CLUs involved in sperm maturation and related to motility was noted [30]. Furthermore, HSPA2 and HSPA9, both of which belong to the heat-shock protein family, were downregulated. Although the function of HSPA9 in fertility is unclear, HSPA2 contributes to sperm identification. HSPA9 is known to function in the regulation of mitochondrial protein folding; therefore, it may be related to regulation of sperm metabolism and energy.

In the present study, we specifically verified the downregulation of ACO2 and AK1, which play important roles in energy metabolism and are vital for sperm motility [13, 14], as well as the upregulation of YBX1, which is a DNA- and RNA-binding protein critical for spermatogenesis [16]. Furthermore, we observed that YBX1 was highly expressed in the sperm head of sperm from patients requiring ICSI treatment, whereas AK1 and ACO2 were downregulated in the sperm head and sperm tail of the same patients, respectively. ACO2 is an important metabolic enzyme of the tricarboxylic acid (TCA) cycle. It plays a role in catalyzing the reversible hydration of cis-aconitic acid to produce citric acid or isocitric acid [31]. Previous evidence supports the significant downregulation of ACO2 expression in patients with asthenozoospermia. Incubating sperm from patients with asthenozoospermia in isocitrate reversed the lack of sperm isocitrate and inhibition of the TCA cycle caused by low ACO2 expression, consequently improving sperm motility [13]. In our study, bioinformatics analysis showed that proteins in the PPI network of ACO2, such as SLC25A3, are involved in energy metabolism and differentially expressed in patients with asthenozoospermia [32]. Therefore, a decline in the level of ACO2 in the sperm of infertile men may lead to abnormal energy metabolism and impaired athletic performance. AK1, an enzyme expressed in the sperm flagella, was also found in the sperm tail in our study [14]. Previous studies have indicated that AK1 regulates the homeostasis of ATP energy and plays a regulatory role in buffering adenine charge in sperm cells [14, 15]. Our PPI network indicated that catenin FH of AK1 is an enzyme involved in the TCA cycle, which suggests that AK1 is associated with the energy-driven motility of sperm and the consequent fertility. YBX1 is an RNA-binding protein and a downstream target of AK1. AK1 directly phosphorylates YBX1 and regulates its ability to bind mRNA, which in turn affects the maintenance, regeneration, and differentiation of spermatogonial stem cells [16, 33]. To date, the role of YBX1 in sperm has not been reported. In our PPI network, YBX1 was observed to be connected to a series of proteins, including MAPK1, which is expressed in the sperm head and could be related to the acrosome reaction of sperm [34]. MAPK inhibitor prohibits sperm energy harvesting [35]. Therefore, we postulate that the upregulation of YBX1 contributes to the downregulation of AK1, abnormal energy metabolism pathways, and subsequent infertility, although a more detailed mechanistic study is required.

There are several limitations to the current study. First, this is a single-center study with a small sample size; therefore, further studies with larger sample sizes will be required to confirm our findings. Second, only three proteins were subjected to further validation, whereas other differentially expressed proteins were not validated. Third, the differentially expressed proteins that were validated, namely AK1, ACO2, and YBX1, were not subjected to further in-depth investigation; thus, their roles and mechanisms concerning infertility remain unconfirmed. Overall, further exploration in these areas will be required.

In summary, the protein expression profiles of sperm from normal fertile men and infertile men with reduced sperm motility and abnormal sperm morphology who required ICSI were compared. The downregulation of ACO2 and AK1 and the upregulation of YBX1 in sperm from infertile males with severe oligoasthenoteratozoospermia requiring ICSI treatment were identified and compared. Although the exact roles of these proteins in infertility and the underlying mechanisms of their action require further study, the findings of this study shed light on the etiology, diagnosis, and potential treatment of male infertility.

## Supplementary Information

Below is the link to the electronic supplementary material.

Supplementary file1 (XLSX 180 kb)

## Data Availability

The datasets generated during and/or analyzed during the current study are available from the corresponding author on reasonable request.
